# Prevalence of hypoalbuminemia and nutritional issues in hospitalized elders**^1^**

**DOI:** 10.1590/1518-8345.0260.2736

**Published:** 2016-08-08

**Authors:** Felipe Brock, Luiz Antonio Bettinelli, Taise Dobner, Júlio César Stobbe, Gabriela Pomatti, Cristina Trevizan Telles

**Affiliations:** 2 MSc, Full Professor, Universidade Regional Integrada do Alto Uruguai e das Missões, Campus de Erechim, Erechim, RS, Brazil.; 3 PhD, Full Professor, Universidade de Passo Fundo, Passo Fundo, RS, Brazil.; 4 MSc, Student of the Multidisciplinary Integrated Health of the Elderly Residency Program and Cander, Universidade de Passo Fundo, Passo Fundo, RS, Brazil.; 5 PhD, Physician, Hospital São Vicente de Paulo, Passo Fundo, RS, Brazil.; 6 Undergraduate student in Nursing, Universidade de Passo Fundo, Passo Fundo, RS, Brazil.

**Keywords:** Hypoalbuminemia, Hospitalization, Aging, Malnutrition, Health of the Elderly

## Abstract

**Objective:**

to estimate the prevalence of hypoalbuminemia in hospitalized elders, related to socio-demographic variables, nutritional status and length of stay.

**Methods:**

crosscutting study with 200 patients hospitalized in a large hospital in the South of Brazil during three months. Evaluations, lab tests and interviews through questionnaires were performed.

**Results:**

the average albuminemia was 2,9 ± 0,5g/dL. Hypoalbuminemia was diagnosed in 173 subjects (87%) and was absent in 27 (13%) that have normal albuminemia (p=0,000). After six days of hospitalization, the prevalence of low levels grew significantly to 90% (p=0,002), average 2,7 ± 0,5g/dL. Using the Mini Nutritional Assessment, it was observed that 41 patients were malnourished and from those, 40 had hypoalbuminemia.

**Conclusion:**

the prevalence of hypoalbuminemia proved to be high, in approx. nine in ten elders, and the nutritional status and the length of stay proved to be related to the decrease of serum albumin levels. Thus, it is suggested that monitoring albumin levels should be done to evaluate the risk that the patient has to develop malnutrition and other complications during hospital stays.

## Introduction 

The Brazilian elderly population grew significantly in the last decades. The average life expectancy of the Brazilian was 74,8 years in 2013. This growth in the population of seniors brings to the fore a new epidemiologic profile for healthcare, and carries in itself a range of modifications and re-signification, as it demands this population to adapt in order to maintain its quality of life^1^ ^-^ ^2^. In the view of these epidemiological shifts, hospitalization is frequently needed to cure pathologies linked to the aging process, among them the circulatory and respiratory being the most prevalent^3^. In those hospitalized elders, a factor that worsen their health status is their nutritional condition, as it affects among others, the tissue regeneration, the inflammatory reaction and the immune function^4^.

The nutritional status is inversely associated to hospitalization and mortality risks. At the same time, the nutritional status deterioration, along with the evolution of the disease itself, may be launched by different factors related to the food supply, being key to assess the status of hospitalized elders, both to prevent malnourishment and to begin an adequate approach and intervention.

For the nutritional assessment of these patients, it is absolutely needed to know the changes that are characteristics of this process, as the progressive loss of lean body mass, and corporeal fluids, the growth in the amount of fat tissue, the reduction of some organs (kidneys, liver and lungs) and the loss of skeletal muscle mass^4^. 

One way of assessing the nutritional status of the patient is through dosage of the serum albumin, that is a biochemical marker widely used in the clinical practice in this area. In geriatric patients, hypoalbuminemia may be physiologic, as the aging process is linked with lower levels of serum albumin, that is 20% lower in individuals over 70 years old. In this population, levels more than 20% under standard may signal protein malnutrition and hypercatabolism, leading to extended lengths of stay, more expensive treatments and implying risks for other kinds of clinical complications^5^.

In spite of the limits imposed by the extended half-life interfering in the detection of acute changes in nutritional status, serum albumin levels are strongly related to morbidity elevations (extension of length of stay, poor healing-up of wounds) and mortality in both acute and chronic disease patients. That is why it is one of the most frequently used variables to compose prognostic indexes, and also the best isolated predictive index of complications. The normal serum albumin concentration is between 3,5g/dL e 5,0g/dL^6^.

The serum albumin concentration depends on many factors such as: hepatic synthesis, hepatocyte function, and ingestion and absorption of protein subtracts; abnormal loss of albumin: kidney disease (Nephrotic syndrome), eclampsia, Protein-losing enteropathy, and burns; high catabolism, infection and distribution volume: affected by hydration status^7^, usual issues in hospitalized patients. That is why malnutrition, the seriousness of diseases, the drugs that are being administered, the length of stay and the age, are important factors to be monitored with regard to the prognosis of the hospitalized elder patient^6^.

Not withstanding this fact, there are scarce Brazilian articles evaluating this tracer in elderly patients. There are no available data in the literature about the prevalence and the clinical significance of the albuminemia in hospitalized patients, and is unknown the extension, the nutritional troubles and the associated factors. Also, the results of this study may support health programs and create protocols to speed-up care to elder population from the beginning of the hospitalization.

In this way, this paper had the objectives of: to estimate the prevalence of hypoalbuminemia in hospitalized elders, related to socio-demographic variables, nutritional status and length of stay.

## Method

This is a prospective crosscutting and analytic study that measured the prevalence of hypoalbuminemia in hospitalized elders in the South of Brazil, between April and June 2012, on a total of 200 elderly patients. The study site is a tertiary level teaching hospital; with a macro-regional catching area, serving approximately two million people of the North of Rio Grande do Sul, West of Santa Catarina and part of Parana, as well as other states of Brazil. It is part of the Brazilian National Health System and has 617 beds.

The number of patients to be interviewed was calculated through a sampling formula, using the following parameters: sample error 5%, level of confidence 95%, population 900 people (average of elders hospitalized each month in the hospital), 84% was the maximum percentage (as per a study performed in 2010^8^. It was needed to have 169 elderly patients in the study plus a 10% for possible losses, a total of 186 individuals to be studied that should conform to the inclusion and exclusion criteria that will be noted below. 

The inclusion criteria were: age equal or more than 60 years old (using as parameter the age classification for elders of the World Health Organization -WHO for developing countries), to agree upon participation and signing the Free and Informed Consent Form (FICF). Exclusion criteria were defined as: seniors that were discharged or deceased in the first 72 hours from hospitalization, permanently in bed, amputations, or not having a responsible person or not being able to sign the FICF, and those that did not receive a serum albumin dosage during hospitalization.

Data collection was done through interview followed by assessment of the clinical records during the first 72 hours of being hospitalized. Data were collected through a previously designed form that evaluated socio-demographic and anthropometric data, clinical information, and lab tests gathered in the hospitalization and six days later. For the assessment the reference values were the usual for the Laboratory of the health facility.

During the interview and to assess the nutritional status of the patient, the Mini Nutritional Assessment (MNA) was used, a questionnaire that comprises 18 items encompassing: anthropometrics, diet evaluation, global clinical evaluation, self-perceived health and nutritional status, that may be used both for triage as well as for assessment. It is a validated and pertinent method for diagnosis of malnutrition and malnutrition risk in elders^9^ ^-^ ^10^.

The values for the classification of Body Mass Index (BMI) were based in the WHO standards in kg/m^2^ for elderly individuals with cutting points different from adults, considering low weight values ≤ 22, eutrophic 22-27 e overweight ≥ 27^11^.

The database was structured using the Microsoft Excel 2007 software, and the analyses were done through the Statistical Package for Social Sciences (SPSS) software, version 20 for Windows. The numeric variables were described with average ± standard deviation and the categorical variables were tested through the Pearson chi-square. The association between albuminemia and quantitative variables were analyzed through Variance analysis. For multiple comparisons was used the post-hoc Tukey test. Results were deemed significant when the association reached values p ≤ 0,05.

The study was approved by the Committee for Ethics in Research of the University of Passo Fundo (UPF) through verdict number 619/2011, respecting resolution 196/1996 of the National Health Council. 

## Results

The participants were 200 elderly inpatients of a hospital in the South of Brazil, in different wards. The average age was 72,6 ± 8,3 years and the majority, 120, were males. The race was white in 89% of the cases the marital status was mainly married or living with a partner and widows, 53% and 37% respectively. The average number of descendants was 3,3± 0,8.

A large part (88%) of the patients were homeowners living in their own houses and belonged to the Catholic religion (90%), 81% were literate, and the average years of schooling was 4,5 ± 3,4. Regarding occupation 88% (n=173) were retired, 19% (n=37) were receiving social security benefits and 3% (n=5) were employees. The albuminemia status of the patients did not present any significant relation with any socio-economic data as recorded in this study.

**Table 1:** Serum albumin at admission and six days after admission in elders, Passo Fundo, RS, Brazil, 2012.

Albumin		Results of albuminemia (n=200)	Average/Standard deviation	p
At admission				0,000
Low		173 (87%)	2,8 ± 0,4	
Normal		27 (13%)	3,7 ± 0,3	
Six days after admission				0,000
	Low	110 (90%)*	2,6 ± 0,5	
	Normal	12 (10%)*	3,7 ± 0,2	

Values express average ± standard deviation or absolute or relative frequency. *Only 122 (61%) patients had a 2^nd^ blood sample for testing albuminemia.

The average albumin level was 2,9 ± 0,5g/dL, and the minimum and maximum values were 1,1g/dL and 4,4 g/dL (Table 1). In 87% (n=173) of the patients a diagnosis of hypoalbuminemia was found and 13% (n=27) had serum albumin levels considered to be in the normal range.

The prevalence of hypoalbuminemia in elders at the sixth day of hospitalization was 90% (n=110) and only 10% (n=12) had normal albumin levels, a statistically significant difference (p=0,000) for both classifications (Table 1). Not all the patients that measured the albumin levels for the first time also had this measure in the second time, a total of 3%, some of them being discharged or dead before the second sampling. The average of albumin dosage in the sixth day was 2,7 ± 0,5g/dL, being the minimum 1,4g/dL and maximum 4,0g/dL. There were no cases of high dosage of albumin.

The patients presented worsening serum albumin levels compared with the first dosage in the sixth day of hospitalization. From 122 tested patients, 106 had a certain degree of hypoalbuminemia at admission and 110 in the sixth day of hospitalization (Table 2). 

**Table 2:** Association of the results of the albuminemia at admission and after six days in elders. Passo Fundo, RS, Brazil, 2012.

Results after six days	Results at admission		Total (n=122)*	p
	Hypoalbuminemia (n=106)	Normal Albumin (n=16)		
Hypoalbuminemia	98 (89%)	12 (11%)	110	0,002
Normal Albuminemia	8 (67%)	4 (33%)	12	

Values express absolute and relative frequencies. *Total (n=122) refers to the total of patients that underwent two albumin tests.

The difference between the averages of the results of tests for serum albumin at admission, proved to be statistically significant when compared with the levels in the samples collected after six days after admission (p=0,002). This analysis only comprised the elders that had both the first and second blood tests.

When the anthropometric population data were evaluated, it was found that weight was on average 70,4 ± 16,7kg, with a minimum of 30 kg and maximum of 124 kg and height was on average 1,66 ± 0,08m, minimum 1,44m and maximum 1,95 m.

Nutritional status of the hospitalized elders showed the following MNA results: 36% (n=73) patients were out of risk for malnutrition, 43% (n=86) were in risk of malnutrition, and 215 (n=41) were malnourished. 

Following the MNA criteria, 98% of the malnourished patients had low albumin level (n=40), and the percentage with normal levels grew according to the improvement of the nutritional level: malnourished 2%, in risk of malnutrition 13% and out of risk for malnutrition 21%. Regarding the subjects with hypoalbuminemia, when the nutritional status of the elder improves, there is a gradual descent of cases: malnourished 98%, in risk for malnutrition 87% and out of risk for malnutrition 79% (Table 3).

**Table 3:** relation between Mini Nutritional Assessment (MNA), Body Mass Index (BMI) and albumin profile in elderly inpatients. Passo Fundo, RS, Brazil, 2012.

Mini Nutritional Assessment		Results of albumin at admission		Total (n=200)	p
		Hypoalbuminemia (n=173)	Normal Albumin (n=27)		
Mini Nutritional Assessment					0,000
	Out of risk for malnutrition	58 (79%)	15 (21%)	73	
	Risk for malnutrition	75 (87%)	11 (13%)	86	
	Malnourished	40 (98%)	1 (2%)	41	
Body Mass Index					0,119
	Low weight	43 (86%)	7 (14%)	50	
	Eutrophic	69 (88%)	9 (12%)	78	
	Overweight	61 (85%)	11 (15%)	71

Values show absolute and relative frequency.

Another anthropometric data that is relevant, the BMI, did not show significant difference when compared in its classification with the results of albumin at admission (p=0,119). As per its results, 36% of the elders were over weighted and form them 85% had hypoalbuminemia (Table 3). The BMI average was 25,5 ± 5,5 with a minimum of 12 and a maximum of 46.

**Figure 1:**
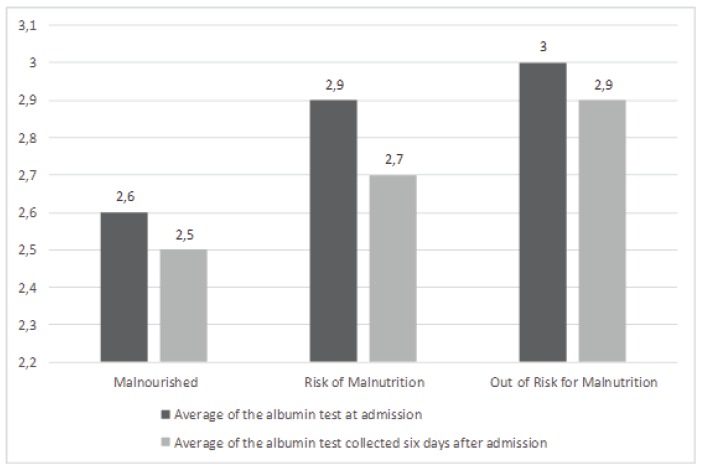
MNA related to average results of albumin test at admission and six days after, in elderly inpatients.

Regarding the first sample for albuminemia tests, the results showed an statistically significant difference from MNA with serum albumin profile (p=0,000) and following the post-hoc Tukey test, there is a difference between the malnourished group and those out of risk of malnutrition (p=0,000). However, this difference was not evident between the group of malnourished and the ones in risk for malnutrition (p=0,092) and between the group out of risk and those in risk for malnutrition (p=0,077). The average level of albumin of malnourished was 2,6 ± 0,5g/dL, of those in risk for malnutrition 2,9 ± 0,5g/dL and those out of risk 3,0 ± 0,4g/dL. The graphic of Figure 1 shows clearly how this relation happens.

Data of albuminemia analysis after six days of hospitalization, also revealed a significant difference between averages, when compared with MNA classification (p=0,009) and through the post-hoc Tukey test, may be perceived a statistically expressive difference only between the malnourished and out of risk groups (p=0,007). The average of the results of the malnourished group, in risk, and out of risk of malnutrition were respectively 2,5 ± 0,6g/dL, 2,7 ± 0,5g/dL e 2,9 ± 0,5g/dL (Figure 1).

## Discussion

This study opened the opportunity for several considerations about the aging process, malnutrition and albuminemia, fostering reflections among health practitioners that provide care for the elder patients. The serum albumin dosage is a easy access marker, has low cost and can stratify the patients by risk during hospitalization, offering at the same time an objective indicator to support decisions when approaching these patients during hospitalization.

Some studies show how hypoalbuminemia is associated with malnourishment^5^ ^,^ ^12^ ^-^ ^14^ and that hospitalization is harmful for it, leading us to the opinion that the longer the hospitalization, larger will be the trend to a higher degree of malnutrition. This situation, ends up launching the serum albumin depletion, as shown in this study, where we can observe that the difference between results were significantly altered between admission and six days after. 

In this paper, hypoalbuminemia may be found in 87% of the studied elders at admission and in 90% after six days, being the averages statistically different (p=0,002). In a research performed in 179 elders hospitalized in a hospital in Brasilia, 84,3% (n=151) of the patients had hypoalbuminemia and an average of 2,74 ± 0,46g/dL, statistically different from the group with normal albumin levels (p=0,001)^8^. Theses results are quite close one to the other, either considering the prevalence or the average, something that helps to confirm in a certain measure, the results of this study. 

Trying to elicit relations between aging and hypoalbuminemia, a longitudinal study was performed in Gifu, Japan in 2007 for five years, evidencing that the levels of serum albumin decreased with age, both in women and men, with significant decline of serum albumin of 0,015g/dL by year in men and 0,012g/dL in women. The relative decrease in those five years was larger in older ages, and reached 1,2% in women with ages between 65-69 years old and 3,1% in those in age group 85-89 (p<0,05)^15^. These findings show that the decline happens progressively, and also evidence the importance of monitoring this population, something that several other authors had already shown in their results^5^.

In another study performed in Japan, analyzing elders in geriatric institutions with the objective of evaluating the relation between serum albumin, anthropometrics and scores of Activities of Daily Life (ADL) and checking at the same time if the albumin level of 3,5 mg/dL as normal for elders, the researchers found that this parameter should not be used in patients with low daily life activities, as it would elevate malnutrition diagnoses, and also that hypoalbuminemia is directly related to the worsening of complications during hospitalization^16^. These facts make evident the need of a complete nutritional assessment of the elderly patient, not only encompassing biochemical markers, but also anthropometrics, food ingest, ADL score and physical check-up, which will make diagnosis more ample and trustworthy. 

The nutritional assessment of the hospitalized patient, and specially of the elder is directed to estimate mortality and morbidity risks by malnutrition, to identify their causes and consequences, in order to guide a nutritional therapy that achieve comprehensive recovery for the patient^17^. It should be done using the adequate tools, and values and ways of testing, that take into account the old age of the patient, paying attention to the loss of autonomy, appetite, vision, olfactory capacity, troubles in chewing, among others^9^. 

Using MNA as the method for assessing nutritional status, the present study found a large number of patients in malnutrition or risk for malnutrition, the same that other study that used the same method when assessing patients in a long-term institution for elders in Guaratinguetá found 28,1% of malnourished patients, 50,6% in risk and 21,3% non-malnourished. These data show that the prevalence of malnutrition and risk of malnutrition is high and represents a public health problem demanding interventions^18^. 

Another study with 92 elders during the first 72 hours of hospitalization in a hospital in Maranhão, revealed that the malnourished patients, according to MNA has depression associated, as assessed by the Geriatric Depression Scale^19^, showing the need of a multiprofessional approach for a comprehensive care of these patients. In Blumenau, SC, 259 patients were assessed after 48 hours from admission in the hospital and from them 49,8% had malnutrition risk and 10,8% were malnourished, being women and people over 75 years the most compromised from the nutritional point of view. The same study found a significant link between BMI and MNA, as the malnourished patients by BMI had 4,7 times more risk of being classified as malnourished by MNA^20^. 

The present research had some limitations, mainly because it was a crosscutting study, not allowing for discrimination of causes and effects. Another limiting factor was the fact that data may be influenced by the circumstance of the elders being in a moment of frailty, that lead to the hospitalization, and it also may launched a more general worsening of the health status, even affecting the lab tests results that were analyzed. 

The results of this study will help to the scientific advancement through an improvement of the knowledge of the albumin profile in relation with nutritional aspects of the elder population. These results also contribute through the proposal of diagnosis models and care for elders presenting hypoalbuminemia and malnutrition that should be applied from the admission moment on, for the elders, society and the work of health practitioners.

## Conclusion 

The prevalence of hypoalbuminemia in hospitalized elders is high and affects approx. nine in ten patients, and the length of stay is linked to the decline in serum albumin levels, not evidenced when related to socio-demographic data.

It was also observed a trend towards a decline of the serum albumin level when the nutritional status is altered, showing a direct link between nutritional and albumin worsening. In this way, if albumin dosage may not be considered a nutritional diagnostic factor, it points to a hike in its risk thus demanding a deeper nutritional diagnosis. It not only is a simple test, but also brings benefits, as it is a lab procedure that may speed-up and improves this diagnosis. 

Other than the albuminemia changes, most elders showed nutritional alterations, and a expressive prevalence of malnourished, or in risk of malnutrition patients. 

Under this general perspective, it is suggested that health practitioners may perform serum albumin level monitoring and the assessment of nutritional malfunction along with the associated factors in elders during hospitalization. This monitoring activity may lead to early interventions to avoid complications such as limb edema, oliguria and pressure ulcers, thus shortening the length of stay and lowering costs.

Finally, it is recommended that longitudinal studies should be performed to assess the serum albumin during hospitalization of elders, receiving or not receiving nutritional support, analyzing its relationship depending upon the length of stay and mortality rates for this population.
